# Sex ratios influence spatial occupancy and kinematic stability of *Anopheles coluzzii* mosquito swarms

**DOI:** 10.1186/s13071-026-07259-7

**Published:** 2026-01-28

**Authors:** Sofia Vielma, Simon P. Sawadogo, Tarwendpanga F. X. Ouédraogo, Antoine Cribellier, Florian T. Muijres, Abdoulaye Diabate, Ruth Müller

**Affiliations:** 1 Unit Entomology, Institute of Tropical Medicine, Antwerp, Belgium; 2 Experimental Zoology Group, Wageningen University, Wageningen, The Netherlands; 3 Institut de Recherche en Sciences de la Santé (IRSS), Bobo-Dioulasso, Burkina Faso; 4 Université Nazi Boni (UNB), Bobo-Dioulasso, Burkina Faso

**Keywords:** *Anopheles coluzzii*, Sex ratio, Mating, Behavioral ecology, Machine learning classification

## Abstract

**Background:**

Malaria mosquitoes reproduce in mating swarms. Previous studies have reported a pronounced activity peak in male mosquito swarms immediately following simulated sunset, typically lasting around 20 min. This peak represents the main swarm formation, where several mosquitoes concentrate above visual markers and maintain prolonged flight activity. However, most studies rely on laboratory setups with balanced or single-sex swarms, which do not reflect the male-biased sex ratios observed in the field.

**Methods:**

In this study, we studied swarming behavior of male and female *Anopheles coluzzii* mosquitoes in five sex ratios (male-only 1:0, male-biased 3:1, balanced 1:1, female-biased 1:3, female-only 0:1) using three-dimensional infrared videography to quantify spatial structure of swarms and flight speed of individual mosquitoes. For each ratio, we analyzed changes in spatial arrangement and flight speed through time and between conditions.

**Results:**

Swarm volume varied following a quadratic trend (${R}^{2}=0.889$). As the proportion of females in the swarm increased, the volume of the swarm increased, ranging from 305.1 cm^3^ in male-biased swarms to 612.6 cm^3^ in female-only swarms. Mean flight speed also increased with female proportion, from $0.66$ m/s (1:1 balanced ratio) to $0.87$ m/s (0:1 female-only ratio), showing a moderate relationship with volume (${R}^{2}=0.504$). Swarm density and speed were negatively correlated, indicating that mixed swarms are not only smaller in volume but also exhibit higher track densities (${R}_{ \mathrm{Spline}}^{2}=0.712)$ suggesting tighter, slower swarms in male-rich groups. Furthermore, we used a Random Forest as an exploratory classifier to (1) identify which kinematic features most differentiate operational sex ratio (OSR) groups and (2) test, as a proof of concept, whether sex ratio can be inferred from kinematic signatures.

**Conclusions:**

These results demonstrate the influence of sex ratio on swarm kinematics and support the use of machine learning for behavioral classification in mosquito ecology.

**Graphical abstract:**

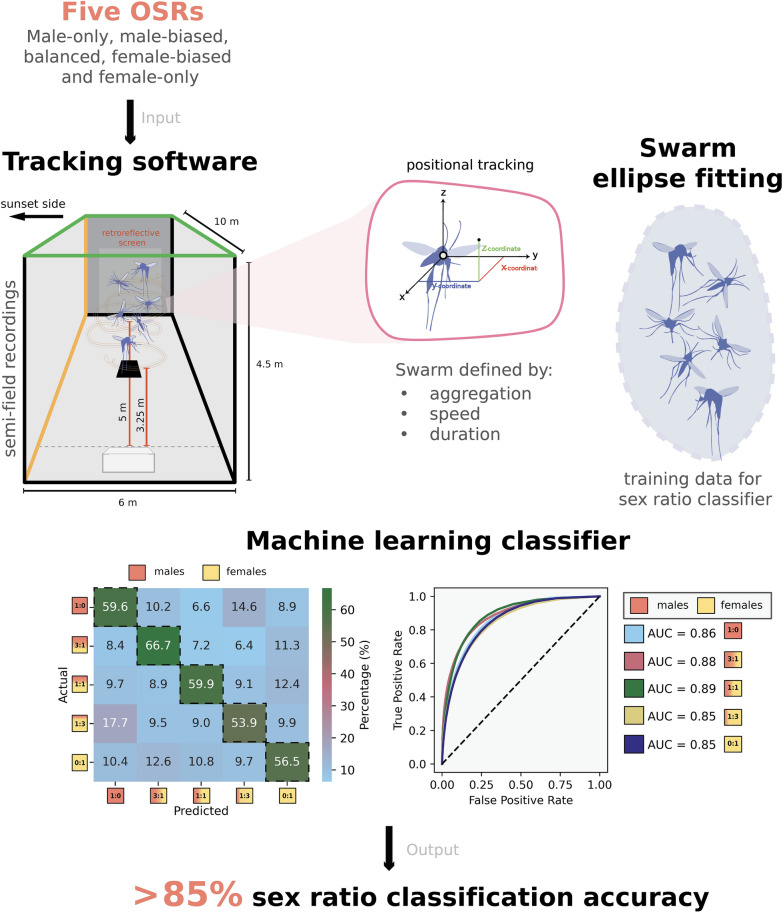

**Supplementary Information:**

The online version contains supplementary material available at 10.1186/s13071-026-07259-7.

## Background

Swarming in *Anopheles gambiae* sensu lato mosquitoes is a unique component of the mating system of this malaria vector, and is characterized by having few to even thousands of individuals flying above swarm markers at sunset and nightfall [1]. The formation of these aggregations can be characterized as a unique chronobiological feature of anopheline mosquitoes where there is suggested to be a high degree of synchrony between male and females [2]. The *Anopheles gambiae* sensu lato species complex comprises several morphologically indistinguishable sibling species [3, 4]. Within this complex, *Anopheles gambiae* sensu stricto and *Anopheles coluzzii* were historically regarded as molecular forms (M and S forms) of a single species. However, subsequent genetic, ecological, and reproductive isolation evidence led to their formal recognition as distinct species following taxonomic revision [5, 6].

As malaria poses an important medical burden, various control approaches have been implemented, yet they mostly relying on decreasing the vector–human contact rather than focusing on the understanding of the behavioral ecology of one of the primary malaria vector in Africa: *Anopheles coluzzii* [7]. Although the ecology of swarms has been extensively studied in the laboratory, semi-field settings, and field settings [8–15], the experimental sex ratios are usually not explicitly controlled or tested. Instead, most laboratory studies rely on balanced (50:50; male:female) groups, while both laboratory and field observations often involve naturally male-biased swarms. Furthermore, although female-biased swarms have been reported in the field for other dipterans [16], there is a lack of behavioral characterization of intermediate sex ratios in laboratory or semi-field studies.

### The potential role of operational sex ratio (OSR) in swarm stability

Operational sex ratio (OSR) refers to the proportion of sexually available males and females in a population [17]. In mosquito swarms, the sex ratio is typically male biased, creating a competitive mating environment where a subset of males achieves reproductive success while others remain unmated [18]. However, the extent to which OSR influences swarm structure, movement dynamics, and mating interactions remains poorly understood. The sex ratio can drive swarming dynamics and shape the evolution of sex roles in sexually reproducing species [19]. In some insects, male-biased swarms can discourage or displace females from forming stable aggregations [20]. In addition, because the number of sexually available females is a limiting factor in mating systems, a strong selection gradient may arise, influencing the development of secondary sexual traits in males and, in some cases, females [21]. For example, increased female ornamentation has been linked to female-biased OSR in other insect species [22]. Furthermore, extrinsic factors such as predation pressure and temperature may also alter sex ratio, further affecting swarm composition and mating success [21].

In *Anopheles gambiae* sensu stricto swarms, a male-biased sex ratio intensifies competition among males, but the strength of sexual selection ultimately depends on how well females can assess potential mates. When assessment is difficult, mating outcomes may become more random, reducing the advantage of sexually selected traits and leaving many males unmated [23]. Conversely, female-biased OSRs are uncommon in mosquitoes and in most insects, but they can occur under specific conditions influenced by local competition, resource availability, or endosymbiont infections [24]. Certain bacteria, such as *Rickettsia*, *Spiroplasma*, and *Wolbachia*, have been suggested as potential drivers of female-biased OSRs by modifying reproductive biology and mating behavior in their hosts, though evidence for this in dipterans remains inconsistent [25]. Despite the recognized influence of OSRs on mosquito mating dynamics, little is known about how different OSRs shape the spatiotemporal dynamics of *Anopheles* swarms, particularly in relation to swarm stability and movement speed. Understanding these relationships is essential for improving predictive models of swarming behavior of *Anopheles gambiae* sensu lato (s.l.) and refining respective vector control strategies and allowing the development of novel vector control tools that leverage OSR-driven effects on swarm stability and mating dynamics.

The present study focuses specifically on *Anopheles coluzzii*, a member of the *An. gambiae* species complex. To study how sex ratios affect swarming behavior of *An. coluzzii*, we conducted three-dimensional flight tracking of swarming mosquitoes at various OSRs in semi-field conditions in Burkina Faso, allowing us to quantify spatial and kinematic parameters and assess their potential for predicting swarm composition using a machine learning framework. For *An. coluzzii*, we hypothesize that: (1) swarm volume is influenced by the proportion of females, (2) mean flight speed alters with the proportion of females, owing to sex-specific flight behaviors, and (3) kinematic parameters such as speed, spatial position, and distance from the swarm centroid can be used to classify swarms by OSR using machine learning. Specifically, we examined whether kinematic swarm stability, defined as the consistency of movement patterns within the swarm, measured through parameters such as speed and spatial position, follows a linear trend with increasing male or female density given that male-only swarms occupy less space than female-only swarms [26], while the spatial dynamics of intermediate sex ratios remain poorly understood. We also investigated the relationship between in-swarm flight speed and OSR, building on observations that swarming females typically fly faster than males [27, 28], to test whether in-swarm flight speed increases in proportion to female density. Finally, we evaluated the extent to which swarm composition can be accurately classified on the basis of movement patterns by integrating kinematic features into random forest classification models, thereby assessing the feasibility of using behavioral metrics for sex ratio prediction. By characterizing the kinematic composition of various operational sex ratios of *An. coluzzii*, we gain insight into potential field conditions that could hinder female-only swarm formation of the malaria vector. This understanding also enables us to identify the most probable swarm scenarios, helping refine mosquito control strategies by aligning interventions with the behavioral signatures of anopheline swarms.

## Methods

### Mosquito rearing and three-dimensional recordings

We performed swarming experiments in semi-field insectaries using *Anopheles coluzzii* mosquitoes (taxonomic serial no. 1151392) obtained from wild gravid females collected in human dwellings in Bama village, Burkina Faso. The strain was established in August 2021 and has been maintained in the insectary at the Institut de Recherche en Sciences de la Santé (IRSS) in Bobo-Dioulasso under local ambient conditions, which during the study period averaged to $27\pm 2$ °C, $80\pm 10\mathrm{\%}$ relative humidity, and $12:12$ L:D light:dark cycle. We reared the larvae and fed them with Tetramin^®^ Baby Fish Food (Tetrawerke, Melle, Germany) *ad libitum*. We removed the pupae daily and sexed them under a stereomicroscope (Leica S6E). We then separated the male and female pupae to ensure virgin status and transferred them into small plastic cups and placed them inside the rearing cages ($30\times 30\times 30$ cm) with approximately 200 individuals each. A total of five different operational sex ratios were generated: 1:0 male-only (200 males), 3:1 male-biased (150 males + 50 females; 200 total), 1:1 balanced (100 males + 100 females; 200 total), 1:3 female-biased (50 males + 150 females; 200 total), and 0:1 female-only (200 females). These classes reflect biologically relevant sex compositions designed to capture a gradient of swarm compositions. For each sex-ratio treatment, biological replication was achieved by testing three independent cohorts of 200 mosquitoes. Each cohort was recorded during a single swarming event, resulting in three recordings per treatment and yielding a total sum of 15 recordings. The replicate sessions were conducted under identical environmental and lighting conditions to minimize recording bias. This design allowed us to systematically explore how changes in sex ratio influence swarm structure and internal flight dynamics and to evaluate whether male- or female-biased groups exhibit distinct spatial or kinematic features. We observed consistent behavioral patterns across replicates and indicate that the dynamics we report are representative of swarms with a given sex ratio.

**Fig. 1:**
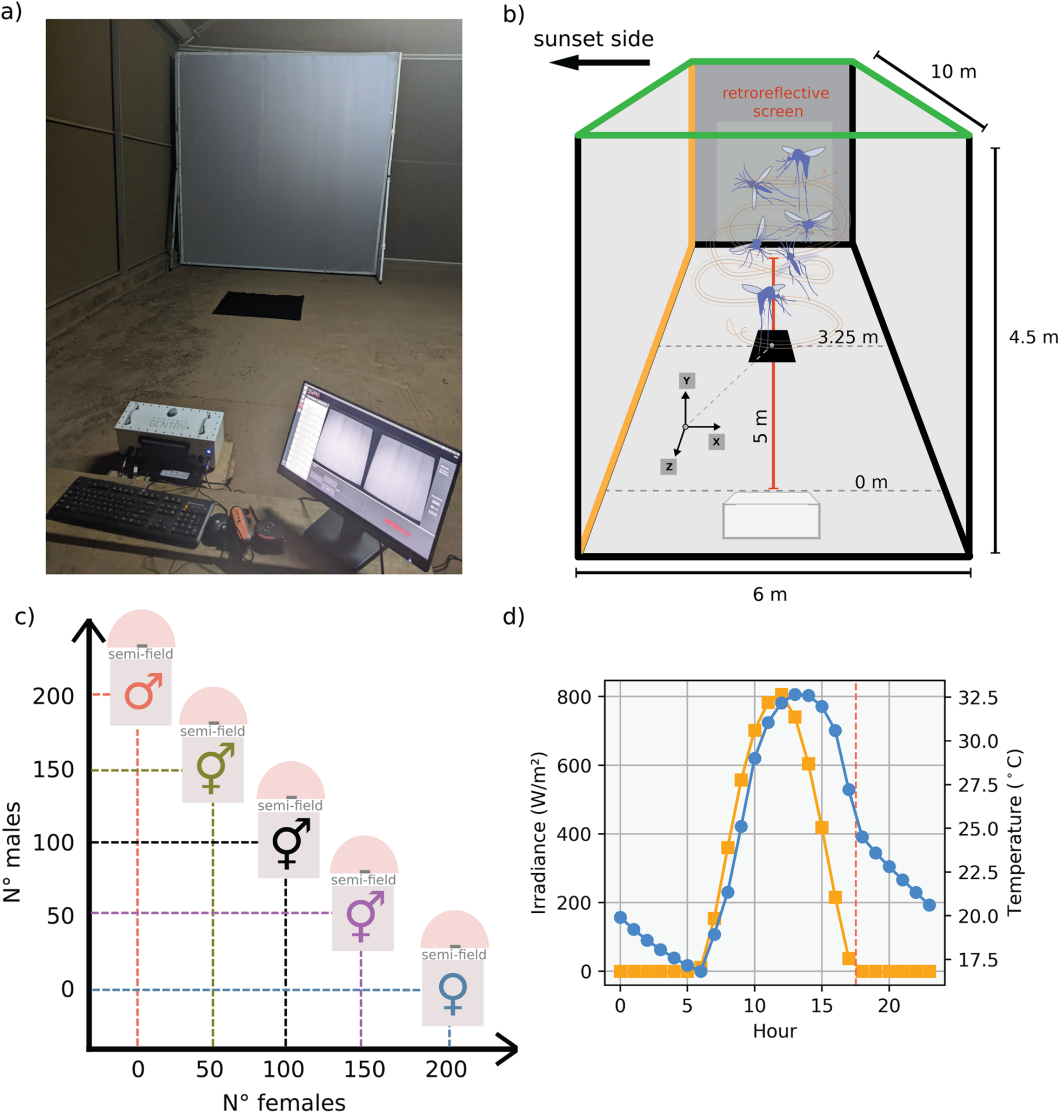
Overview of the experimental setup for mosquito swarming recordings. **a** Photograph of the recording setup, featuring the retroreflective screen and Photonic Fence Monitoring Device used for tracking mosquito flight in three dimensions. **b** Diagram of the experimental arena, illustrating the placement of the recording camera $325$ cm from the swarm marker and the defined tracking area above it. **c** Matrix of tested sex ratios, including male-only (1:0), male-biased (3:1), balanced (1:1), female-biased (1:3), and female-only (0:1), used to assess the effects of sex ratio on swarming behavior. **d** Hourly averages of irradiance (W/m^2^, left axis) and ambient temperature (°C, right axis) across the experimental period in Bobo-Dioulasso. Data were extracted from NASA POWER reanalysis products, showing a sharp peak in irradiance around midday and corresponding gradual increase in temperature, which aligns with typical mosquito swarming time windows shortly after sunset (indicated by red dashed line at 17:52:00)

After emergence, we fed adult mosquitoes with a $10$% glucose solution and kept them in the insectary under local ambient conditions. As the mating status of a female mosquito has a direct influence on its likelihood to swarm (unpublished data by Vielma et al.), adult females were not blood fed before the experiments, ensuring that all individuals remained in a physiological state compatible with swarm formation and avoiding suppression associated with mating or blood feeding. After 5 days, we transferred the mosquitoes to the semi-field setup to prepare for the experiments. Once adults reached sexual maturity, we performed the experiments by tracking the swarming mosquitoes using three-dimensional videography-based recordings with the photonic fence monitoring device (PFMD). This was carried out by placing a camera at a $325$-cm distance from the center of a $100$ × $100$-cm black visual marker in the ground (Fig. 1a, b).

All recording sessions for the different sex ratio treatments (Fig. 1c) were conducted in the mosquito ecology research facility (MERF) located in Bobo-Dioulasso, Burkina Faso, originally described by [29]. The MERF is a large semi-field system specifically designed to enable mosquito behavioral observations under near-ambient outdoor environmental conditions. The facility consists of a single structure composed of two parallel rows of six compartments (12 compartments in total), each measuring 10.0 × 6.0 × 4.5 m (L × W × H). Compartments are separated by a central corridor and enclosed by polyester mesh walls that permit unrestricted airflow, allowing temperature, humidity, and wind conditions inside the compartments to closely track those of the surrounding environment. The roof is made of a thin transparent polyethylene thermal film (Polyane^®^, Agripolyane, France), which allows natural light transmission and diffusion while limiting excessive temperature peaks. The structure is oriented along a north–south axis to ensure homogeneous sky illumination at sunset.

Experiments were performed in a single selected compartment within this structure, with all groups and replicates randomized and conducted in the same large semi-field compartment to ensure consistency across treatments. No artificial lighting, photoperiod manipulation, or active temperature or humidity control was applied in the experimental compartment. Consequently, mosquitoes were exposed to naturally varying dusk light levels and meteorological conditions, particularly during the short period when swarming behavior occurs. Weather conditions during the experimental period (4-week period, from 22 November to 22 December 2022) corresponded to the early dry season and were generally stable. Experiments were conducted only on evenings without rainfall or strong winds, as such conditions are known to disrupt mosquito flight and swarming behavior. Natural day-to-day variation in cloud cover contributed to variability in ambient light intensity at sunset and was considered part of the ecological realism of the semi-field setup.

The irradiance and temperature profiles shown in Fig. 1d were derived from the NASA POWER reanalysis database [30] and are provided to illustrate the natural outdoor environmental cycles prevailing during the experimental period, rather than to represent environmental conditions artificially imposed within the semi-field enclosure. Given the open and ventilated design of the MERF, these outdoor profiles are considered representative of the environmental conditions experienced by mosquitoes inside the enclosure during the experiments.

### Spatial and temporal filtering of data

The Photonic Fence Monitoring Device produced arrays of 3D flight tracks at a temporal resolution of 100 recording frames per second, in the camera frame of reference. First, we transformed this reference frame into the world reference frame in which the marker was located at the origin of the coordinate system ([*X*, *Y*, *Z*] = [0, 0, 0] m). Here, the *X* axis was positioned normal to the sunset horizon, the *Y* axis vertically up, and the *Z* axis parallel to the sunset horizon (Fig. 1).

To further process these tracking data, we applied a series of filters. First, we used a temporal filter to retain only data around the sunset period. Second, we applied a spatial filter to isolate the area of interest above the swarm marker by restricting flight data along both the $X$ axis and *Z* axis to a range of $[-100, 100]$ cm. As shown in Fig. 1b, the PFMD camera was oriented so that the enclosure walls did not enter the active tracking volume. Any potential edge noise from the mesh was further minimized by the spatial filtering step, which restricted the dataset to the central swarm hotspot above the marker and excluded peripheral regions near the enclosure boundaries. Third, we applied a speed filter to reduce noise from potential non-swarming tracks, such as mosquitoes engaging in exploratory flight or edge-associated movement. Previous studies have reported swarm-associated flight speeds averaging between $0.50$ and $0.59$ m/s, which characterize a high-speed movement around the swarm core, whereas low-speed trajectories (< 0.50 m/s) typically correspond to non-swarming behaviors, including exploratory flights near the enclosure edges or brief transitions into the swarm. These slow tracks are short-lived and do not form part of the stable, repetitive flight loops that define swarm participation [26, 31]. On the basis of this evidence, we applied a conservative lower-speed threshold of $0.50$ m/s, excluding any tracks with average speeds below this value while maintaining meaningful biological interpretation of swarms. Finally, we applied a filter on the basis of track duration. Here, we estimated the total flight time of each mosquito and defined swarming individuals as those with a track duration exceeding $25$ s, consistent with previous definitions [31].

### Definition of track-level kinematic features

Each track represents the full three-dimensional flight trajectory of a single mosquito continuously detected by the PFMD system within a recording. For track-level kinematic analyses and classification, individual mosquito features were summarized over the full duration of each track. Specifically, mean flight speed was calculated as the time-averaged speed across all frames belonging to a track. Spatial coordinates (*X*, *Y*, and *Z*) were retained as instantaneous positions for volumetric modeling, while distance from the swarm center was computed as the Euclidean distance from the marker origin and used as a derived spatial descriptor. All kinematic variables reflect averages or distributions across sustained swarming trajectories.

### Data processing for temporal swarm activity analyses

We estimated the temporal dynamics of swarm activity using the total number of swarming mosquitoes within 1-min intervals. We did so by adding up the mosquito counts per min and dividing this by the total number of recording frames per min (6000 at a frame rate of $100$ fps). This approach assumes that each mosquito is counted once per recording frame and that the total number of frames accurately represents the observation period.

### Data processing for volumetric and speed analysis

To quantify the swarm’s occupied volume during this period, we fitted a Gaussian mixture model (GMM) with a confidence ellipsoid over the tracks of each experimental group. This aimed to capture regions with the highest clustering density while following the structural swarm shape defined in previous articles [31–33]. Conceptually, we chose a GMM, since it assumes that the swarm can be represented as a probability distribution composed of one or more Gaussian components [34], each describing how individual mosquitoes are spatially distributed around a central mean position. The model estimates the probability that each data point (mosquito position) belongs to the fitted Gaussian distribution. This ensures that the resulting ellipsoid captures the overall spatial extent and density distribution of the swarm rather than fitting an arbitrary geometric shape to the data (i.e., convex hull). This approach allowed us to estimate the distribution of the tracks within the aggregate in a three-dimensional space and help with identifying the central clustering core. For each replicate of an experimental group, we extracted the distributions of the $X$, $Y$, and $Z$ position of the swarming mosquitoes throughout their flight path and fitted a single component GMM using a full covariance matrix. With this model, we were able to generate a confidence ellipsoid with a $90$% confidence level, using the chi-squared critical value to scale the ellipsoid axes. The Mahalanobis distance was then computed to differentiate the inner core of the swarm from peripheral tracks. The tracks within the ellipsoid (Additional file 1: Supplementary Fig. S2) were considered part of the central cluster.

Once the swarm aggregations were fitted, the total volume inside the ellipsoid was calculated using the product of its principal axes, and we calculated the trend in volume variation in respect of the sex ratio of the experimental group. This was done through curve fitting, using a quadratic function to model the relationship between sex ratio and swarm volume. Furthermore, in addition to volume calculation, the speed (m/s) of each mosquito was also extracted from the inside of the plotted ellipsoid, and a spline interpolation was fitted to estimate the relationship between volume and speed. In addition, we used both a generalized linear mixed model (GLMM) and the spline interpolation to explore the relationship between swarm density and flight speed, since the GLMM tests the overall relationship while accounting for variation between replicates, and the spline captures potential nonlinear patterns in the data. To assess whether the spline model would overfit the data, we compared its performance with linear, quadratic, cubic, and logistic models using the Akaike information criterion (AIC). These predefined curve fits were used as exploratory tools to examine potential nonlinear relationships between swarm volume and internal flight speed. Our goal was not to derive a predictive model or engage in stepwise model selection but rather to assess which general relationship shapes were most supported by the data. The use of AIC allowed us to compare the relative fit of biologically plausible models, and results were interpreted descriptively rather than as formal statistical inference. We acknowledge the importance of avoiding overfitting [35] and emphasize that these models were intended to inform hypotheses, not confirm them. All analyses were conducted using scipy, scikit-learn, numpy, pandas, and matplotlib.

### Exploratory random forest classification to identify OSR-derived kinematic signatures

In addition to extracting kinematic parameters from inside the fitted swarm ellipsoids, we proceeded to isolate the data points from within the inner structure of the ellipsoid and evaluate whether a classification algorithm could be applied to predict the operational sex ratio as the output label. We used a random forest primarily as an interpretive tool to illuminate which kinematic features differentiate swarms across OSR groups and assess the separability among the test groups. We extracted parameters such as flight speed (m/s) and spatial coordinates (*X*, *Y*, and *Z*) and performed feature engineering by using the Euclidean distance from the swarm center (distance from origin = $d$), defined by the following Eq. (1):

1 $$d=\sqrt{{X}^{2}+{Y}^{2}+{Z}^{2}}$$

to capture movement trends. The PFMD system outputs several additional variables (track ID, timestamp, size estimate, acceleration, and a raw distance field), but not all were included in the model because size estimates fluctuate with orientation and lighting, acceleration values are highly noisy, and distance is fully determined by the (*X*, *Y*, and *Z*) coordinates. Therefore, our final feature set consisted of speed (m/s); *X*, *Y*, and *Z* positions; and the derived Euclidean distance $d$, representing all meaningful kinematic parameters relevant to swarm behavior. Once parameters were attained, we randomly divided the dataset into training (80%) and test (20%) sets. Because each mosquito track constituted one observation for the classifier rather than each swarm, the number of available tracks differed substantially among OSR groups (e.g., fewer tracks in female-biased and female-only swarms). This variation led to uneven class representation in the training dataset. To mitigate the imbalance across sex ratio categories in our classification dataset, we applied the synthetic minority oversampling technique (SMOTE) to the training set prior to model fitting. As mentioned previously, our aim was not to derive a predictive model but to explore whether swarm features differed systematically between sex ratio groups. In addition, although random forest classifiers are generally robust to class imbalance, SMOTE helped to provide more balanced representation across OSRs, enabling more informative interpretation of feature importance and classification structure. The technique was limited to the training set to prevent data leakage. We acknowledge that alternatives such as class weighting exist, but opted for SMOTE given its utility in visualizing class separability in ecological datasets [36–39]. We then tested a random forest (RF) model, which we trained with 30 decision trees and that exhibited the best performance in handling complex decision boundaries in high-dimensional spaces. The final feature set was encoded, and categorical labels representing different sex ratios were transformed into numerical values for easier handling (male-only = 0, male-biased = 50, balanced = 100, female-biased = 150, and female-only = 200) before model training. The trained classifier was evaluated using a confusion matrix, precision-recall metrics, F1-score, and receiver operating characteristic (ROC) curves with the area under the curve (AUC) to assess its ability to distinguish between different swarm compositions.

## Results

### Temporal and spatial swarming trends

During our experiments, sunset time occurred approximately at $17:52:00$(hour:minute:second). For all experiments, swarming started approximately 10 min after sunset, followed by a peak in swarm activity period of approximately 20 min (from $18:04:00$ to $18:24:00$) occurring during late sunset and nightfall.

**Fig. 2:**
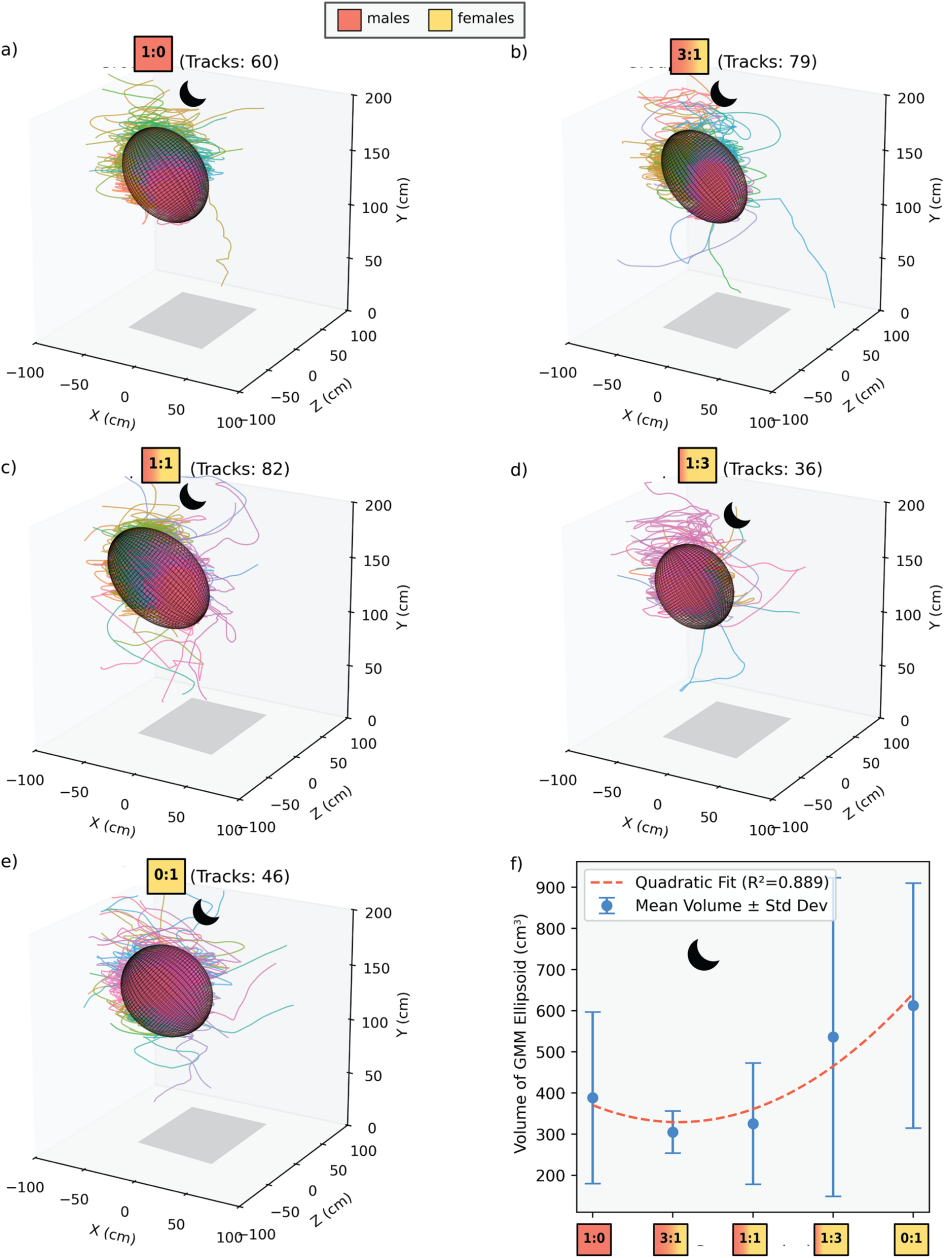
Ellipsoid volume fitting per experimental group. The volume (${\mathrm{cm}}^{3}$) for each group with standard deviation bars was calculated and quadratic fit (dashed red line) illustrates the nonlinear relationship between swarm volume and sex ratio, with the largest volumes observed in female-biased and female-only swarms (${R}^{2}=0.889$). The observed increase in volume with higher female presence suggests that sex ratio influences swarm structure, with mixed groups showing intermediate volume expansion. Ellipsoids were fitted using Gaussian mixture models (GMM) on 3D mosquito flight tracks. Volumes were computed from the principal axes of each fitted ellipsoid using custom Python scripts (see https://github.com/svielma96/PFMD-Gaussian-model)

During the peak swarming period, the three replicates of each of the five experimental groups yielded a total 60 swarming tracks for the 1:0 male-only group (20.00 ± 13.45 tracks per swarm; Fig. 2a), 79 for the 3:1 male-biased group (26.33 ± 22.47 tracks per swarm; Fig. 2b), 82 for the 1:1 balanced group (27.33 ± 15.50 tracks per swarm; Fig. 2c), 36 tracks for the 1:3 female-biased group (12.00 ± 12.16 tracks per swarm; Fig. 2d), and lastly, 46 tracks for the female-only experimental groups (15.33 ± 13.31 tracks per swarm; Fig. 2e).

**Fig. 3:**
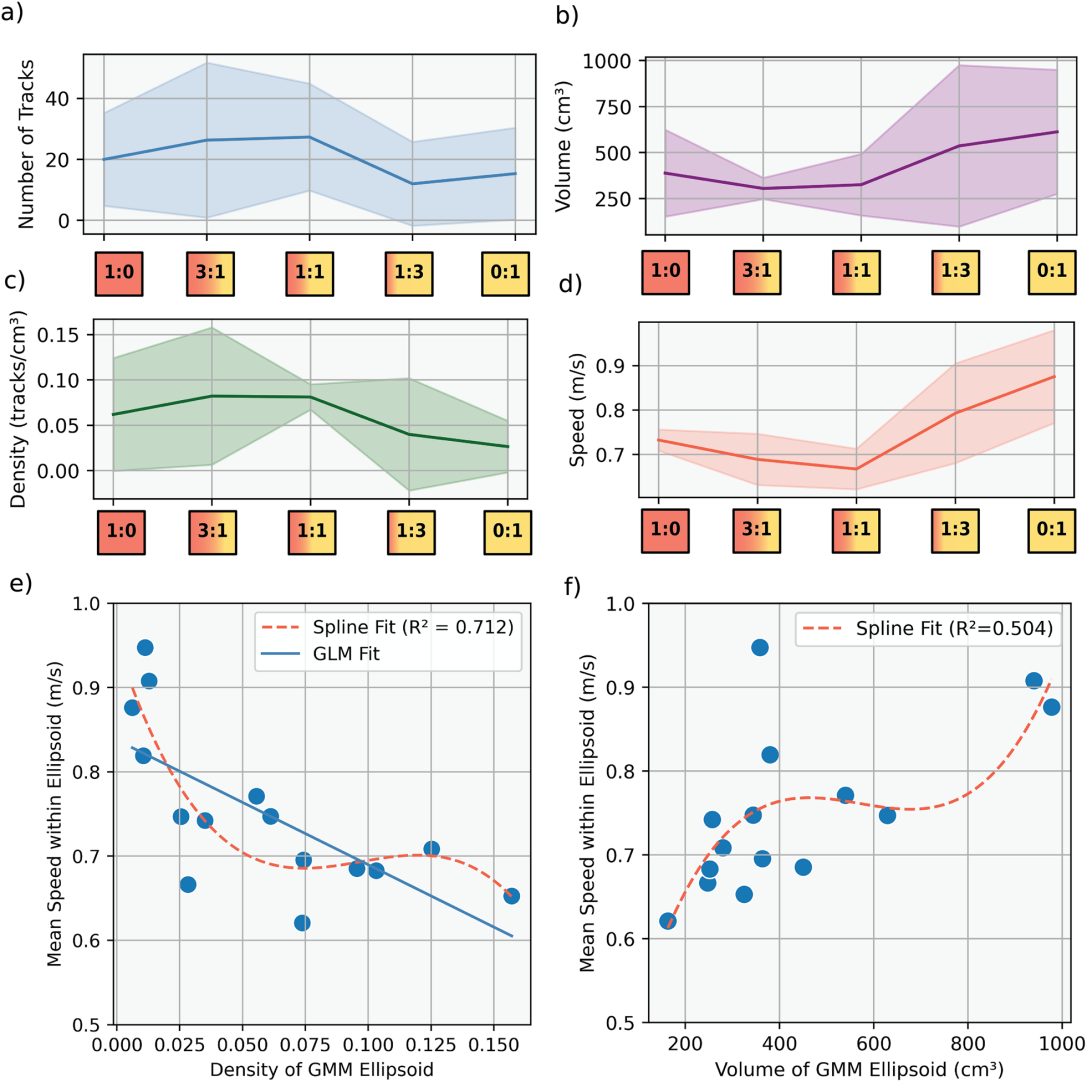
Kinematic and spatial metrics of *Anopheles coluzzii* swarms across sex ratio groups. **a** Mean number of tracks per swarm within the fitted ellipsoid with 95% confidence intervals (CI), indicating the highest track counts in male-biased and balanced swarms. **b** Mean flight speed within fitted ellipsoids with 95% CI. Female-biased and female-only groups exhibit the highest speeds. **c** Swarm density, calculated as track count per ellipsoid volume (tracks/cm^3^), is the highest in male-rich swarms and decreases with increasing female proportion, with 95% CI. **d** Swarm volume, represented by the 90% confidence ellipsoid around each swarm, increases in female-biased and female-only groups (95% CI shown). **e** The relationship between ellipsoid volume and mean flight speed. A third-order spline fit (dashed red line) indicates a nonlinear relationship (*R*^2^ = 0.504), where speed increases with volume but stabilizes at higher swarm sizes. **f** The relationship between swarm density and mean speed. Both a spline fit (dashed red line) and GLM fit (solid blue line) indicate a strong negative correlation (*R*^2^ = 0.712), where increasing density is associated with decreased flight speed

Having established differences in the number of swarming individuals across operational sex ratios, we next examined how these differences translate into swarm-level spatial structure. The fitted ellipsoid swarm volumes did not follow a linear trend with respect to OSR (Figs. 2f, 3a). The measured ellipsoid volumes vary across the different group compositions, with group 1:0 (male-only) showing $388.4\pm 208.5$ cm^3^, group 3:1 (male-biased) reaching $305.0\pm 50.9$ cm^3^, group 1:1 (balanced) peaking at $325.5$ ± 147.5 cm^3^, group 1:3 (female-biased) increasing to $536.3$ ± 387.3 cm^3^, and group 0:1 (female-only) exhibiting the largest swarm volume at $612.6\pm 297.7$ cm^3^ (Fig. 3b). These variations suggest that aggregation size is influenced by factors beyond sex ratio alone. Furthermore, we applied a curve fitting that follows a quadratic polynomial trend characterized by the equation:

2 $$V\left(x\right)=a{x}^{2}+bx+c,$$

where $x$ represents the operational sex ratio and $V(x)$ is the estimated swarm volume. The optimized model parameters obtained through nonlinear least squares regression were $a=0.0146, b=-1.5524,\text{ and }c=370.4547,$ yielding the final quadratic equation as $V(x)=0.0146{x}^{2}-1.5524x+370$.4547.

The quality of the fit was evaluated using the coefficient of determination (${R}^{2}$), which was $0.889$, indicating a strong correlation between sex ratio and swarm volume. The results indicate a nonlinear relationship between sex ratio and swarm volume, supporting the hypothesis that swarm structure is influenced by the composition of males and females.

### Negative relationship between swarm density and flight speed

We estimated the relative swarm density (Fig. 3c) as the ratio between the number of flight tracks in each swarm and the corresponding swarming volume (number of tracks per given volume). Here, the 1:0 male-only group density was 0.06 ± 0.05 tracks per cm^3^, the 3:1 male-biased density was 0.08 ± 0.06 tracks per cm^3^, the 1:1 balanced group density was 0.08 ± 0.01 tracks per cm^3^, the 1:3 female-biased group density was 0.03 ± 0.05 tracks per cm^3^, and for the female-only experimental groups, the density was 0.02 ± 0.02 tracks per cm^3^. This shows that the female-dominated swarms had the lowest density, caused by both the low number of swarming tracks and the large swarming volume. In contrast, the 3:1 male-biased and 1:1 balanced groups had the highest densities, caused by the combination of high swarming numbers and small swarming volume. Based on the AIC criterion, the spline model yielded the lowest AIC (−93.25), indicating the best balance between model complexity and fit (Additional file 1: Supplementary Fig. S3a). We used a generalized linear mixed model (GLMM) capturing the fixed effect of density. The model revealed a significant negative association between density and speed (*β* = − 1.4789 ± 0.412, *p* < 0.001), with an intercept of 0.837 and a pseudo-*R*^2^ of 0.581. In addition, we combined this test with a spline interpolation fit to model the speed-to-density relationship (Fig. 3e), given by the equation:

3 $$S\left(x\right)=\sum_{i=1}^{n}{\alpha }_{i}{B}_{i}\left(x\right),$$

where ${B}_{i}(x)$ are the basis functions and ${\alpha }_{i}$ are the corresponding coefficients determined via smoothing constraints. Furthermore, on the basis of the GLMM model, we can present the relationship between our parameters as follows:

4 $$\widehat{y}= 0.8376 - 1.4789 \times \mathrm{density}$$

where $\widehat{y}$ is the predicted mean speed (m/s) and density is the independent variable. The coefficient of determination (${R}^{2}$), which evaluates the model performance, indicates a moderately high predictive power (${R}_{ \mathrm{Spline}}^{2}=0.712,{R}_{ \mathrm{GLMM}}^{2}= 0.581)$.

### Nonlinear relationship between swarm volume and flight speed

The quadratic fit indicates a nonlinear relationship between sex ratio and swarm volume, with larger volumes observed in female-biased groups. While this pattern may reflect shifts in aggregation behavior, we interpret it as descriptive rather than indicative of a defined threshold. To further examine this pattern, we analyzed how mean flight speed varied across sex ratio groups (Fig. 3d) and how flight speed related to swarm volume (Fig. 3f). The mean speed of mosquitoes within each fitted ellipsoid was compared with its corresponding estimated volume, aiming to assess whether a behavioral shift is associated with swarm size. To model this relationship, we tested a spline model (See Eq. 3). On the basis of the AIC criterion, the third-order spline model yielded the lowest AIC (−78.85), indicating the best balance between model complexity and fit (Additional file 1: Supplementary Fig. S3b). For this model, the fitting quality was assessed using the coefficient of determination (${R}^{2}$), which yielding moderate predictive power (${R}^{2}=0.504$). The relationship between speed and swarm volume appeared nonlinear, with speed generally increasing alongside volume but showing signs of stabilization in larger aggregations. Notably, for large swarms (> 700 cm^3^) the flight speeds tend to further increase.

The observed trend reflects a nonlinear pattern in aggregation dynamics, as swarms with greater volume tend to display more dispersed and variable flight trajectories compared with smaller, tightly clustered swarms. Furthermore, the density plots of volume and speed reveal a bimodal distribution, suggesting the presence of two distinct behavioral states within the swarm. This further supports the idea that swarm dynamics are influenced by different movement strategies or structural organization depending on aggregation size.

### Random forest-based classification of swarm sex composition

The classification process is based on a random forest algorithm, which aggregates predictions from multiple decision trees using majority voting according to

5 $$\widehat{y}=\frac{1}{T}\sum_{t=1}^{T}{h}_{t}\left(x\right),$$

where $\widehat{y}$ is the predicted class, $T$ is the total number of decision trees, and ${h}_{t}(x)$ represents the prediction from each individual tree for input $x$. The importance of each feature is determined using the mean decrease in impurity (MDI) as

6 $$I\left( f \right) = \mathop \sum \limits_{t = 1}^{T} \mathop \sum \limits_{{n \in N_{t} }} \Delta i_{n} \cdot 1\left( {f_{n} = f} \right),$$

where $I(f)$ quantifies the contribution of feature $f$ in reducing classification uncertainty. This analysis highlights that speed, height, and spatial positioning are key factors in differentiating swarm compositions.

**Fig. 4:**
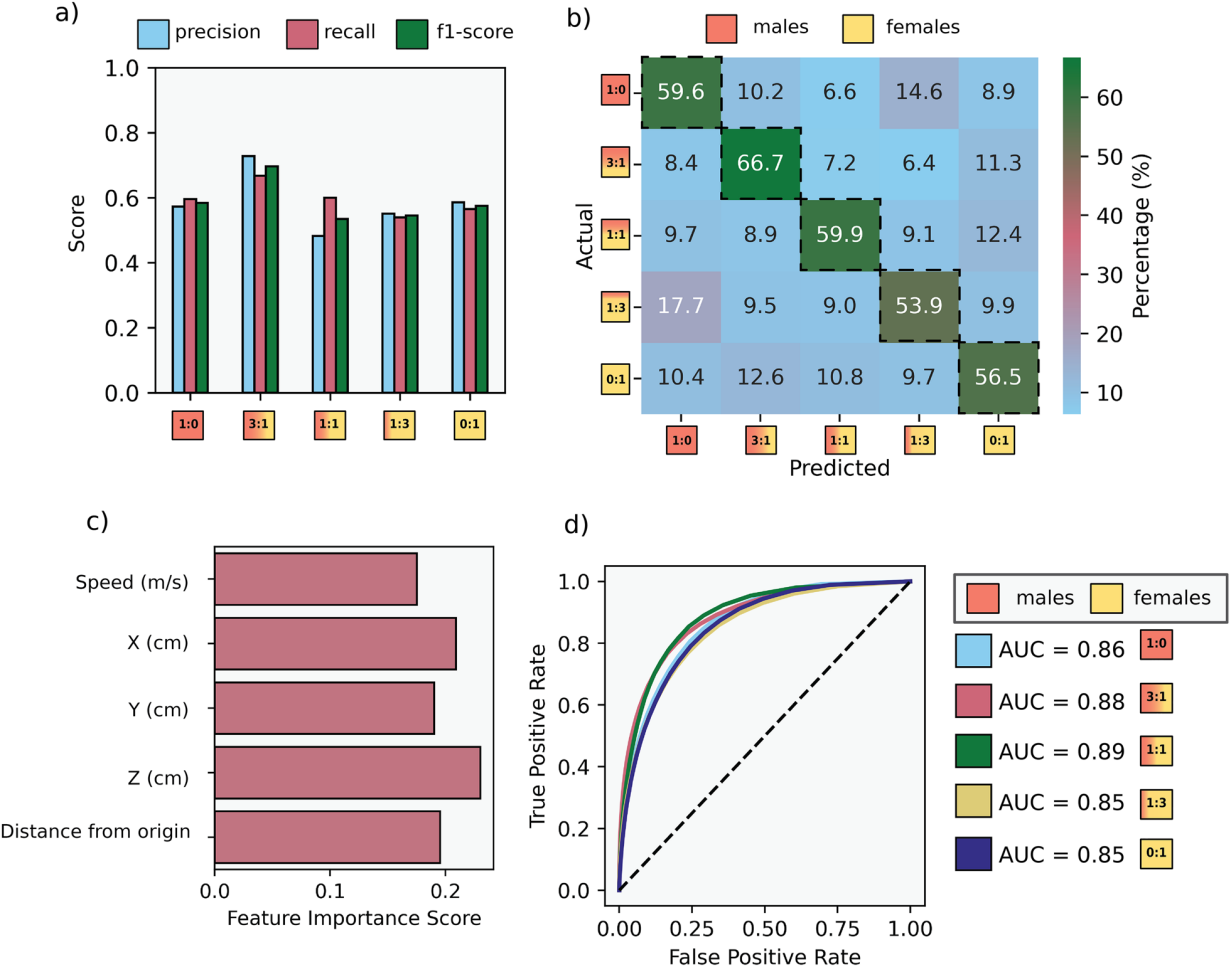
Modeling of operational sex ratio using a random forest classifier. **a** The classification performance metrics are characterized by precision, recall, and *F*1 score across different sex ratios displaying that the model achieves a consistent performance across groups. **b** The highest accuracy is observed in the male-biased (3:1), male-only (1:0), and balanced (1:1) groups, with $66.7$%, $59.7$%, and $59.7$% accuracy, respectively, as shown in the confusion matrix. **c** Feature importance analysis ranking the contribution of different movement parameters to classification shows position over the marker ($X,Y,Z$) is the most influential feature when making the classifier. **d** The AUC values exceed $0.8$ for most groups, showing a strong predictive performance

The overall *F*1 score shows that the classifier is generally robust across different sex ratios (Fig. 4a). The confusion matrix demonstrates that the model correctly classifies the majority of observations, with the highest accuracy observed in the male-biased (3:1), male-only (1:0), and balanced (1:1) groups, which achieved $66.7\mathrm{\%}, 59.7{\% and }59.7{\% accuracy}$, respectively (Fig. 4b). Intermediate groups, such as the balanced (1:1) and female-biased (1:3) groups, showed slightly lower classification performance, suggesting overlapping movement patterns between sexes in mixed groups. Feature importance analysis highlights that height ($Y$), spatial positioning ($X,Z$), and distance from origin are the most important kinematic components for classification (Fig. 4c). Our ROC curves indicate strong model discrimination, with AUC values above 0.85 for all groups, confirming that the model effectively differentiates swarm compositions (Fig. 4d).

## Discussion

Using three-dimensional videography-based tracking and a machine learning modeling framework, we were able to characterize trends in the swarm volumes and flight speeds of *Anopheles coluzzii* swarming and predict varying OSRs. Our results suggest that the sex ratio has a strong influence on swarm dynamics, as seen by the comparison between male-biased and female-biased swarms. Female-dominated swarms had fewer swarming individuals, encompassed a larger volume, and consequently had lower swarming densities. In contrast, the male-biased swarms consisted of larger numbers and were more compact (lower volume and higher track densities). Strikingly, in the complete absence of females, the 1:0 male-only group produced more dispersed swarms with numbers and densities intermediate between the female-biased and male-biased swarms. Interestingly, the relationship between swarm volume and sex ratio follows a quadratic trend rather than a simple linear increase toward male or female dominance. The track count and density data presented in Table 1 can help explain this: swarms that are male-biased contain many swarming individuals, leading to compact aggregations with high density and lower volume. Conversely, when females dominate, the aggregation size increases but the number of individuals remains low, leading to loose swarms with low density. This may suggest that male swarming activity is strongly stimulated by female presence, while the reverse effect is weaker, adding a few males to a female swarm does not lead to the same increase in aggregation. Moreover, we show that mixed swarms fly at lower speeds than male-only and female-dominated swarms.

**Table 1:** Mean and standard deviation (SD) of swarm volume and average flight speed for each experimental group (*n* = 3 experiments per group)

OSR group	Number of tracks		Volume (cm^3^)		Density (track/cm^3^)		Speed (m/s)	
Male:female ratio	Mean	SD	Mean	SD	Mean	SD	Mean	SD
1:0 (Male-only)	20.00	13.45	399.8	248.8	0.06	0.05	0.73	0.02
3:1 (Male-biased)	26.33	22.47	302.1	58.5	0.08	0.06	0.68	0.05
1:1 (Balanced)	27.33	15.50	327.4	152.3	0.08	0.01	0.66	0.04
1:3 (Female-biased)	12.00	12.16	553.3	380.7	0.03	0.05	0.79	0.09
0:1 (Female-only)	15.33	13.31	625.3	283.5	0.02	0.02	0.87	0.05

These combined results suggest three distinct swarm strategies due to males modifying their swarming behavior in response to female presence: (1) a compact, tight-knit aggregation in male-biased groups, (2) a loose, spatially expansive configuration in female-dominated swarms, and (3) an intermediate behavior in male-only swarms. This behavioral response of increased swarming activity in denser aggregations might increase the chance of swarming males to successfully reproduce with the females present. In contrast, when females dominate the swarm, this tight clustering seems to relax, resulting in more dispersed swarm structures.

### Characterization of swarming activity across various sex ratios

These patterns suggest that swarm expansion is limited by changes in aggregation density and participation rather than scaling directly with increasing male or female representation. Moreover, this nonlinear relationship between sex ratio and swarm volume did not translate into a direct relationship between swarm size and individual flight speed. Although it would be expected that a larger swarm could allow for more dispersal and increased flight space [10, 12], one might expect a proportional increase in flight speed as mosquitoes navigate within a more spacious swarm, hinting toward a linear relationship. However, our analysis suggests that this effect is not strongly linear, as indicated by the spline fit of speed versus swarm volume, which shows only a relatively average correlation between these variables. The observed relationship between swarm density and speed also supports a mechanistic explanation grounded in maneuverability and energetics. Compact, high-density swarms require more frequent and tighter turns, which constrain movement and lower overall flight speed. Looser aggregations, in contrast, permit higher speeds, potentially owing to lower navigational demand [40]. Therefore, the effect of swarm size on flight speed is not purely volumetric but mediated by how spatial constraints shape movement effort. As previously reported, mosquitoes may be attracted to the swarm center by speed-dependent forces, so whenever encountered with adverse environmental conditions, mosquitoes may respond by increasing their swarm speed and become more tight-knit [33]. In dense, tight-knit swarm structures, males likely engage in increased competition to secure a mate [7]. This competition may drive males to position themselves advantageously within the swarm, optimizing their chances of locating and successfully mating with a female. A potential advantage of this dense grouping may relate to the predator confusion hypothesis, where it has been suggested that the aggregation of several individuals moving in a swarm could potentially confuse an approaching predator [41]. While this has been proposed in other taxa and supported in systems involving alarm calls or coordinated movement [42], its applicability to mosquitoes remains speculative and requires empirical validation. Although plausible, there is currently no direct evidence supporting aerodynamic benefits in mosquito swarms. This should be framed as a testable hypothesis in future studies involving controlled wind tunnel or computational fluid dynamics analyses. However, the observation that swarming flight consumes over 50% of available sugar glycogen reserves [43] underscores the high energetic demand of swarming and highlights its direct impact on reproductive success and survival [44]. In contrast, female-biased swarms exhibit looser aggregation patterns, which may not function as competitive arenas like their male-dominated counterparts. Instead, females could hypothetically be maximizing male attraction rather than competing directly for mates. These findings align with previous reports describing a swarm core surrounded by chaotic, dispersed flight trajectories [26]. Such behavior may serve as a type of “offering flight” strategy, where females remain in a more dynamic spatial state, potentially scanning multiple swarm sites until a mating event occurs [45]. The notion that loose aggregation reduces predation risk is another speculative interpretation though plausible, this idea needs experimental substantiation. A notable example is dragonflies (*Odonata*), which are known to prey on mosquitoes. In many cases, a single large mosquito swarm may contain approximately $0.2$ g of biomass, making tightly packed aggregations more attractive to predatory dragonflies [46].

Since our results suggest distinct spatial patterns across sex-biased swarms, we aimed to quantify and test these differences using a random forest machine learning classifier. This approach allowed us to identify unique OSRs on the basis of kinematic features such as speed, height, and spatial positioning. It must be noted that our goal was not to develop a black-box predictive machine learning (ML) model but rather to explore ecological relationships between swarm structure and internal flight dynamics. Machine learning tools are increasingly used not to generate predictions alone but to gain ecological insight from large datasets [47, 48]. Preprocessing steps such as filtering and time correction were necessary to ensure biological plausibility and data quality [31]. Thus, the random forest model was employed as a complementary classification tool to identify multivariate differences across sex ratio treatments, not as an end-to-end predictive model. Our findings indicate that male-biased swarms were classified with higher accuracy, likely owing to their tight-knit composition. In contrast, female-biased and mixed groups showed greater overlap, suggesting higher behavioral variability in female-dominated swarms compared with male-dominated ones. Interestingly, the chaotic and complex nature of swarm formations appears to contribute to aggregation robustness. In our analysis, we utilized fitted ellipsoids to capture swarm spatial properties; however, this raises an important question: what extrinsic factors influence the elliptical shape of these aggregations, and to what extent does directionality contribute to their stability? [49]. In short, positions contribute to the classification of sex ratios owing to males and females exhibiting different spatial strategies during swarming: males cluster tightly around the marker to maximize mating opportunities, while females tend to fly more loosely and explore a broader space. These sex-specific aggregation patterns create distinct spatial signatures that our classifier uses to distinguish swarm compositions.

Furthermore, although mating events were not directly quantified in this study, previous work has shown that mating success in *Anopheles* swarms depends strongly on spatial proximity, encounter rate, and flight pairing between males and females [11, 50–52]. A compact, high-density swarm would allow an increase in encounter frequency and competitive interactions among males, thereby enhancing opportunities for successful copulation. Conversely, a looser and more spatially dispersed aggregation may reduce encounter rates and weaken competitive dynamics. The kinematic metrics quantified here as swarm volume, density, and internal flight speed, therefore represent behavioral proxies that are mechanistically linked to mating opportunity rather than mating success per se. By demonstrating systematic differences in these metrics across operational sex ratios, our results provide a behavioral framework through which these ratios may indirectly shape reproductive outcomes in mosquito populations.

Beyond their role in mating, swarm shape has been noted to emerge from an interplay between intrinsic behaviors and environmental cues, where relative position to the sunset plays a major role in directionality and swarm structure. Mosquitoes have been theorized to use an “optical marker angle” parallel to the horizon to locate themselves above a swarm marker [31]. Given these findings, predicting swarm structures through machine learning has the potential to enhance vector control strategies, particularly for interventions aimed at disrupting mosquito mating behavior. For instance, the more dispersed and variable organization of female-biased swarms suggests that inducing similar dynamics in male swarms could reduce swarm stability and, consequently, lower encounter rates and mating efficiency.

### Limitations and variability

While we acknowledge that in some cases the inter-replicate variability in the number of detected swarming tracks was substantial, such variability is expected when working with live, free-flying insects in semi-field conditions. In these scenarios, stochastic environmental effects can strongly influence participation in swarming behavior. Consequently, as previously mentioned, patterns reported here should be interpreted as descriptive trends rather than precise quantitative estimates. While the observed relationships between sex ratio, swarm volume, density, and speed were consistent across replicates, additional experiments with increased replication will be necessary to assess the generality and robustness of these patterns across populations and environmental contexts.

### Using machine learning approaches for vector control

Although various machine learning techniques have been implemented for mosquito vector control by leveraging the distinct characteristics of different mosquito life stages, visual-based algorithms face significant challenges owing to the small size of mosquitoes. This limitation highlights the need for further research into mosquito ecology and behavioral biology, particularly swarming behavior, to enhance detection and intervention strategies [53]. However, integrating machine learning models with existing surveillance technologies could enable automated swarm identification and classification in field conditions [54, 55], allowing interventions to be planned on the basis of detected sex ratios. Since male swarms tend to be tightly knit, whereas female-dominated swarms are more chaotic and loosely structured, understanding these differences could provide insight into why females do not form dense clusters and whether inducing such clustering could aid intervention efforts. It has been suggested that females exhibit greater swarm marker exploration rather than forming cohesive aggregations [26]. Understanding the behavioral mechanisms governing these differences could inform the development of density-based trapping strategies, where mosquitoes are artificially directed toward a marker to form a high-density cluster, allowing for a targeted control approach. Although purely theoretical at this stage, continued research into the behavioral ecology of *Anopheles* swarms could provide valuable insights for the development of novel malaria control tools.

## Supplementary Information

**Additional file 1.**

## Data Availability

All data used in this study were generated from semi-field recordings using the Photonic Fence Monitoring Device (PFMD). The raw data related to the present study are openly available via The Open Science Framework: https://osf.io/6nkyq/. Experimental recordings were processed using the Gaussian-based swarm structure model described in the article. The full analysis pipeline, including data cleaning, 3D Gaussian Mixture Model fitting, and model fitting scripts, is available on GitHub: https://github.com/svielma96/PFMD-Gaussian-model.git. The repository is licensed under the Apache License, version 2.0.
