# Supplementary material for: Sex ratios influence spatial occupancy and kinematic stability of Anopheles coluzzii mosquito swarms
Source: Parasit Vectors. 2026 Jan 28;19:54. doi: 10.1186/s13071-026-07259-7 (PMC12849574; doi:10.1186/s13071-026-07259-7)
Supplement: Supplementary file 1 — Additional file 1. [file 13071_2026_7259_MOESM1_ESM.docx]

**Supplementary Material.**


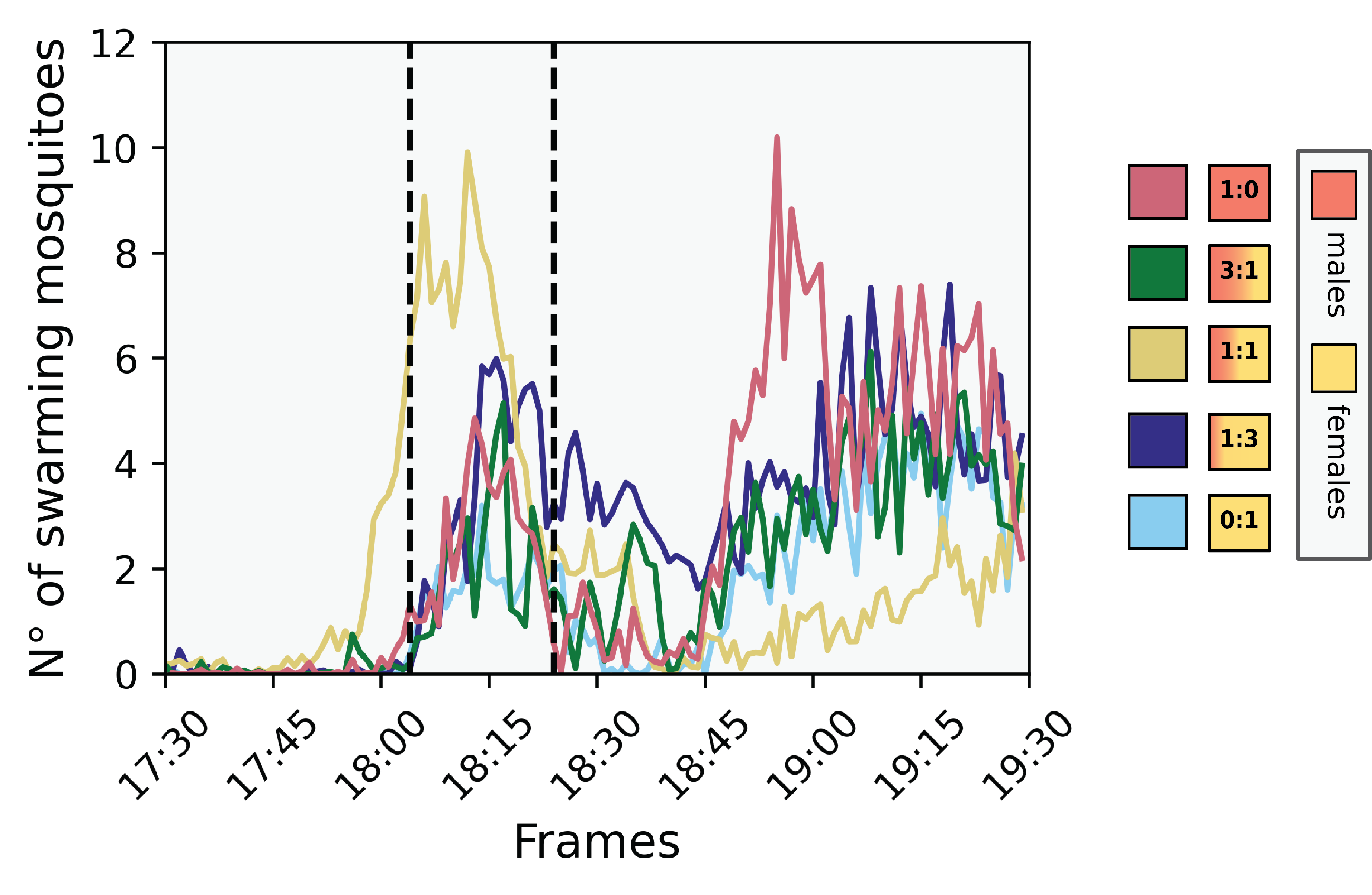


**Supplementary Fig. 1. Sex ratio activity pattern of mosquitoes over sunset and night.** Line plot depicts the distinct behavioral pattern in both male and female mosquitoes over time, ranging from daylight to night. There is a considerable increase in the total number of detected individuals during "swarming peak" time which is not constant across time.


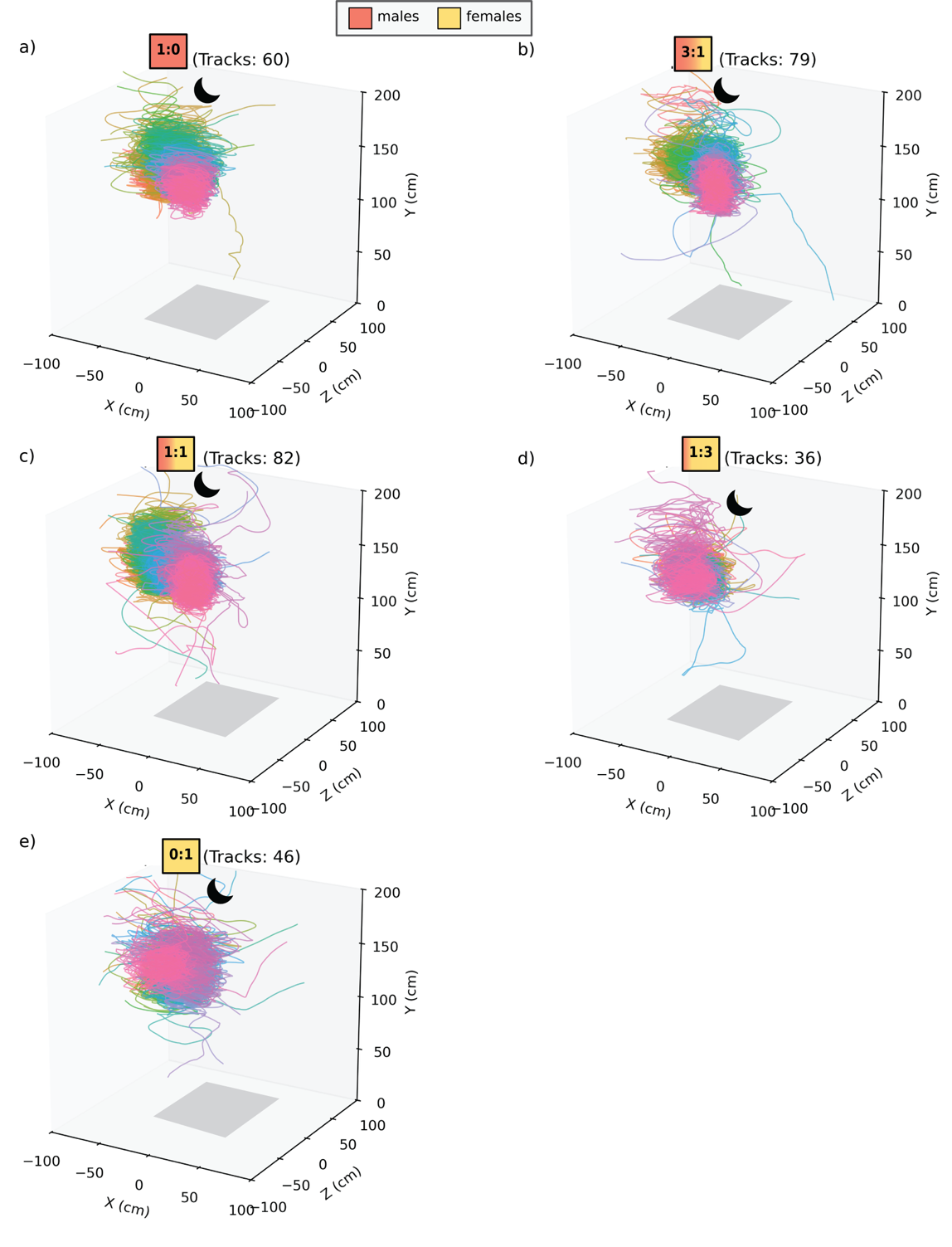


**Supplementary Fig. 2. Raw 3D flight trajectories used for ellipsoid volume fitting across sex ratio conditions.** Each coloured line represents the full flight trajectory of an individual mosquito track within a recording. Trajectories are shown in the world reference frame relative to the swarm marker (grey square). Panels correspond to different operational sex ratios (indicated in the upper left of each panel), with the total number of tracks shown for each condition. These raw trajectories illustrate differences in swarm spatial extent across sex ratios, over which the Gaussian ellipsoid model was subsequently fitted for volumetric analysis.


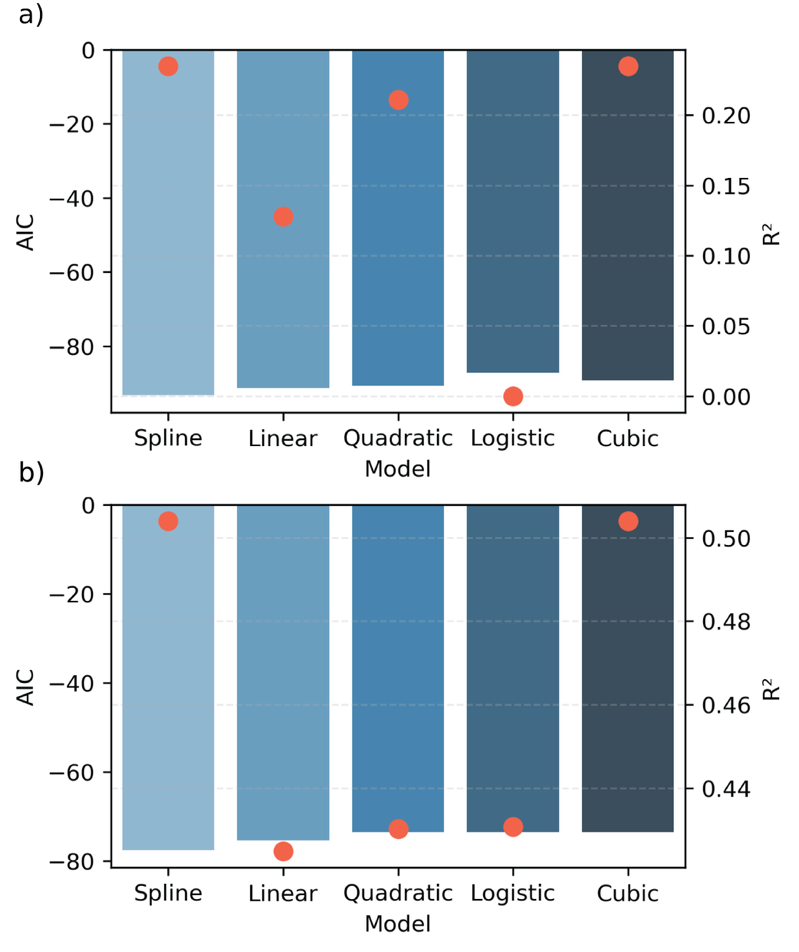


**Supplementary Fig. 3. Model performance comparison for fitting the relationship between kinematic parameters**. (a) Tests the relationship between swarm speed and density and (b) tests the relationship between speed and volume. In both cases we used five different models: spline, linear, quadratic, logistic, and cubic. The height of the blue bars depicts the Akaike Information Criterion (AIC), with lower values indicating a better balance of model fit and complexity. The red dots indicate the R² values for each model. Among all models, the spline fit achieved the lowest AIC and the highest R², suggesting it offers the best trade-off between predictive accuracy and complexity. While the cubic model reached a similar R², its higher AIC indicates potential overfitting.
